# 6-Amino-3-methyl-4-(4-nitro­phen­yl)-1-phenyl­pyrazolo[3,4-*b*]pyridine-5-carbonitrile

**DOI:** 10.1107/S1600536808006156

**Published:** 2008-03-12

**Authors:** Xin-Ying Zhang, Xiao-Yan Li, Xia Wang, Xue-Sen Fan, Gui-Rong Qu

**Affiliations:** a School of Chemical and Environmental Sciences, Henan Key Laboratory for Environmental Pollution Control, Henan Normal University, Xinxiang, Henan 453007, People’s Republic of China

## Abstract

The title compound, C_20_H_14_N_6_O_2_, contains four rings. The dihedral angle between the pyridine ring and the pyrazole ring is 1.9 (1)°, *i.e.* almost coplanar, which gives rise to a conjugated structure. The dihedral angle between the nitro-substituted phenyl ring and the pyridine ring is 76.3 (1)° and that between the pyrazole ring and the non-substituted phenyl ring is 40.5 (1)°. In the crystal structure, symmetry-related mol­ecules are linked by N—H⋯O and C—H⋯N hydrogen bonds.

## Related literature

For related structures, see: Quiroga *et al.* (1999 ▶); Zhu *et al.* (2005 ▶). For the biological and pharmacological activities, see: Kamal *et al.* (1991 ▶); Straub *et al.* (2001 ▶); Sekikawa *et al.* (1973 ▶).
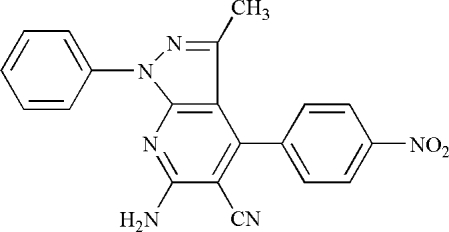


## Experimental

### 

#### Crystal data

C_20_H_14_N_6_O_2_
*M* *_r_* = 370.37
Monoclinic, 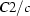

*a* = 16.470 (11) Å
*b* = 9.742 (7) Å
*c* = 23.46 (2) Å
β = 105.857 (8)°
*V* = 3621 (5) Å^3^
*Z* = 8
Mo *K*α radiation
μ = 0.09 mm^−1^
*T* = 294 (2) K
0.39 × 0.25 × 0.15 mm

#### Data collection

Bruker SMART CCD area-detector diffractometer
Absorption correction: multi-scan (*SADABS*; Sheldrick, 1996 ▶) *T*_min_ = 0.945, *T*_max_ = 0.986
13443 measured reflections
3374 independent reflections
2287 reflections with *I* > 2σ(*I*)
*R*_int_ = 0.027

#### Refinement

*R*[*F* ^2^ > 2σ(*F* ^2^)] = 0.042
*wR*(*F* ^2^) = 0.121
*S* = 1.02
3374 reflections
254 parameters
H-atom parameters constrained
Δρ_max_ = 0.18 e Å^−3^
Δρ_min_ = −0.16 e Å^−3^

### 

Data collection: *SMART* (Bruker, 2007 ▶); cell refinement: *SAINT* (Bruker, 2007 ▶); data reduction: *SAINT*; program(s) used to solve structure: *SHELXS97* (Sheldrick, 2008 ▶); program(s) used to refine structure: *SHELXL97* (Sheldrick, 2008 ▶); molecular graphics: *SHELXTL* (Sheldrick, 2008 ▶); software used to prepare material for publication: *SHELXTL*.

## Supplementary Material

Crystal structure: contains datablocks I, global. DOI: 10.1107/S1600536808006156/su2046sup1.cif

Structure factors: contains datablocks I. DOI: 10.1107/S1600536808006156/su2046Isup2.hkl

Additional supplementary materials:  crystallographic information; 3D view; checkCIF report

## Figures and Tables

**Table 1:** Hydrogen-bond geometry (Å, °)

*D*—H⋯*A*	*D*—H	H⋯*A*	*D*⋯*A*	*D*—H⋯*A*
N5—H5*A*⋯O2^i^	0.86	2.13	2.981 (3)	168
C14—H14⋯N2^ii^	0.93	2.61	3.529 (3)	168

Symmetry codes: (i) 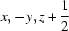; (ii) 
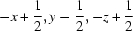
.

**Fig. 1.**
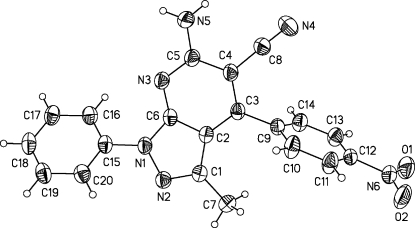
Molecular structure of the title compound, with displacement ellipsoids drawn at the 30% probability level.

## supplementary crystallographic information

### Comment

The structure of the title compound, (I), is shown below. Dimensions are available in the archived CIF.

For related literature, see Quiroga *et al.* (1999); Zhu *et al.* (2005). For the biological and pharmacological activities, see Kamal *et al.* (1991); Straub *et al.* (2001); Sekikawa *et al.* (1973).

### Experimental

The title compound was prepared by the following procedure: To 1 ml of 1-butyl-3-methylimidazolium tetrafluoroborate ([bmim][BF_4_]) were added 4-nitrobenzaldehyde (1 mmol), malononitrile (1 mmol) and 5-amino-3-methyl-1-phenylpyrazole (1 mmol). The reaction mixture was stirred at 80°C for 10 hrs. The yellow solid product that was obtained was collected by suction and rinsed with water and ethanol (yield 93%). Single crystals of the title compound were obtained by slow evaporation from ethanol.

### Refinement

H-atoms were included in calculated positions and treated as riding atoms: N—H = 0.86 Å and C—H = 0.93 - 0.96 Å with *U*_iso_(H) = 1.5*U*_eq_(CH_3_) and 1.2*U*_eq_(NH_2_,CH).

### Crystal data

**Table:**

C_20_H_14_N_6_O_2_	*F*_000_ = 1536
*M**_r_* = 370.37	*D*_x_ = 1.359 Mg m^−^^3^
Monoclinic, *C*2/*c*	Mo *K*α radiation λ = 0.71073 Å
Hall symbol: -C 2yc	Cell parameters from 2825 reflections
*a* = 16.470 (11) Å	θ = 2.5–21.6º
*b* = 9.742 (7) Å	µ = 0.09 mm^−^^1^
*c* = 23.46 (2) Å	*T* = 294 (2) K
β = 105.857 (8)º	Block, yellow
*V* = 3621 (5) Å^3^	0.39 × 0.25 × 0.15 mm
*Z* = 8	

### Data collection

**Table:**

Bruker SMART CCD area-detector diffractometer	3374 independent reflections
Radiation source: fine-focus sealed tube	2287 reflections with *I* > 2σ(*I*)
Monochromator: graphite	*R*_int_ = 0.027
*T* = 294(2) K	θ_max_ = 25.5º
φ and ω scans	θ_min_ = 2.5º
Absorption correction: multi-scan(SADABS; Sheldrick, 1996)	*h* = −19→19
*T*_min_ = 0.945, *T*_max_ = 0.986	*k* = −11→11
13443 measured reflections	*l* = −28→28

### Refinement

**Table:**

Refinement on *F*^2^	Secondary atom site location: difference Fourier map
Least-squares matrix: full	Hydrogen site location: inferred from neighbouring sites
*R*[*F*^2^ > 2σ(*F*^2^)] = 0.042	H-atom parameters constrained
*wR*(*F*^2^) = 0.122	*w* = 1/[σ^2^(*F*_o_^2^) + (0.0546*P*)^2^ + 1.3917*P*] where *P* = (*F*_o_^2^ + 2*F*_c_^2^)/3
*S* = 1.02	(Δ/σ)_max_ < 0.001
3374 reflections	Δρ_max_ = 0.18 e Å^−^^3^
254 parameters	Δρ_min_ = −0.16 e Å^−^^3^
Primary atom site location: structure-invariant direct methods	Extinction correction: none

### Special details

**Table:**

Geometry. All e.s.d.'s (except the e.s.d. in the dihedral angle between two l.s. planes)are estimated using the full covariance matrix. The cell e.s.d.'s are takeninto account individually in the estimation of e.s.d.'s in distances, anglesand torsion angles; correlations between e.s.d.'s in cell parameters are onlyused when they are defined by crystal symmetry. An approximate (isotropic)treatment of cell e.s.d.'s is used for estimating e.s.d.'s involving l.s. planes.
Refinement. Refinement of *F*^2^ against ALL reflections. The weighted *R*-factor *wR* andgoodness of fit *S* are based on *F*^2^, conventional *R*-factors *R* are basedon *F*, with *F* set to zero for negative *F*^2^. The threshold expression of*F*^2^ > σ(*F*^2^) is used only for calculating *R*-factors(gt) *etc*. and isnot relevant to the choice of reflections for refinement. *R*-factors basedon *F*^2^ are statistically about twice as large as those based on *F*, and *R*-factors based on ALL data will be even larger.

### Fractional atomic coordinates and isotropic or equivalent isotropic displacement parameters (Å^2^)

**Table:**

	*x*	*y*	*z*	*U*_iso_*/*U*_eq_	
C1	0.14291 (11)	0.3249 (2)	0.22385 (8)	0.0539 (5)	
C2	0.13161 (11)	0.17956 (19)	0.22657 (8)	0.0508 (4)	
C3	0.12907 (11)	0.06720 (19)	0.18900 (8)	0.0514 (5)	
C4	0.11731 (12)	−0.0608 (2)	0.21136 (8)	0.0579 (5)	
C5	0.10829 (12)	−0.0751 (2)	0.27046 (8)	0.0586 (5)	
C6	0.11882 (11)	0.1536 (2)	0.28254 (8)	0.0515 (5)	
C7	0.16473 (14)	0.4076 (2)	0.17675 (9)	0.0675 (6)	
H7A	0.1171	0.4109	0.1424	0.101*	
H7B	0.2117	0.3663	0.1665	0.101*	
H7C	0.1795	0.4992	0.1910	0.101*	
C8	0.11094 (16)	−0.1792 (2)	0.17454 (10)	0.0771 (7)	
C9	0.13676 (11)	0.08314 (19)	0.12762 (8)	0.0516 (5)	
C10	0.06928 (13)	0.1307 (3)	0.08327 (9)	0.0750 (7)	
H10	0.0187	0.1510	0.0919	0.090*	
C11	0.07559 (14)	0.1485 (2)	0.02644 (9)	0.0749 (7)	
H11	0.0300	0.1808	−0.0034	0.090*	
C12	0.15017 (13)	0.11769 (19)	0.01483 (8)	0.0573 (5)	
C13	0.21832 (13)	0.0685 (2)	0.05755 (9)	0.0656 (6)	
H13	0.2684	0.0471	0.0484	0.079*	
C14	0.21111 (12)	0.0516 (2)	0.11416 (8)	0.0617 (5)	
H14	0.2568	0.0184	0.1437	0.074*	
C15	0.11107 (11)	0.3128 (2)	0.36614 (8)	0.0539 (5)	
C16	0.14851 (12)	0.2328 (2)	0.41484 (9)	0.0610 (5)	
H16	0.1792	0.1550	0.4107	0.073*	
C17	0.13988 (13)	0.2697 (2)	0.47001 (9)	0.0674 (6)	
H17	0.1640	0.2155	0.5029	0.081*	
C18	0.09585 (14)	0.3862 (3)	0.47639 (10)	0.0732 (7)	
H18	0.0910	0.4111	0.5136	0.088*	
C19	0.05918 (14)	0.4654 (2)	0.42781 (11)	0.0722 (6)	
H19	0.0294	0.5440	0.4322	0.087*	
C20	0.06610 (12)	0.4293 (2)	0.37226 (10)	0.0636 (5)	
H20	0.0408	0.4828	0.3394	0.076*	
N1	0.12015 (10)	0.27857 (17)	0.30914 (7)	0.0569 (4)	
N2	0.13518 (10)	0.38354 (17)	0.27275 (7)	0.0592 (4)	
N3	0.10738 (10)	0.03211 (17)	0.30563 (6)	0.0575 (4)	
N4	0.1045 (2)	−0.2721 (2)	0.14393 (10)	0.1197 (9)	
N5	0.10113 (12)	−0.20117 (18)	0.29226 (8)	0.0784 (6)	
H5A	0.0962	−0.2102	0.3276	0.094*	
H5B	0.1015	−0.2726	0.2708	0.094*	
N6	0.15858 (14)	0.14160 (19)	−0.04520 (8)	0.0750 (5)	
O1	0.22406 (12)	0.1080 (2)	−0.05634 (7)	0.0962 (6)	
O2	0.09960 (14)	0.1956 (2)	−0.08097 (7)	0.1125 (7)	

### Atomic displacement parameters (Å^2^)

**Table:**

	*U*^11^	*U*^22^	*U*^33^	*U*^12^	*U*^13^	*U*^23^
C1	0.0489 (10)	0.0631 (12)	0.0496 (11)	0.0009 (9)	0.0132 (8)	0.0005 (9)
C2	0.0476 (10)	0.0611 (12)	0.0439 (10)	0.0024 (8)	0.0128 (8)	−0.0009 (9)
C3	0.0479 (10)	0.0626 (12)	0.0425 (10)	0.0093 (9)	0.0102 (8)	0.0001 (9)
C4	0.0672 (13)	0.0611 (12)	0.0430 (10)	0.0062 (9)	0.0111 (9)	−0.0021 (9)
C5	0.0655 (12)	0.0633 (13)	0.0455 (11)	−0.0049 (10)	0.0128 (9)	−0.0017 (9)
C6	0.0499 (11)	0.0610 (12)	0.0439 (10)	−0.0028 (9)	0.0135 (8)	−0.0063 (9)
C7	0.0721 (13)	0.0687 (13)	0.0632 (13)	−0.0008 (11)	0.0210 (10)	0.0070 (11)
C8	0.1150 (19)	0.0602 (14)	0.0533 (13)	0.0113 (13)	0.0184 (12)	0.0015 (11)
C9	0.0545 (11)	0.0572 (11)	0.0428 (10)	0.0114 (9)	0.0128 (8)	−0.0004 (8)
C10	0.0606 (13)	0.1155 (19)	0.0501 (11)	0.0348 (12)	0.0173 (10)	0.0073 (12)
C11	0.0726 (14)	0.1049 (18)	0.0449 (11)	0.0368 (13)	0.0120 (10)	0.0089 (11)
C12	0.0760 (13)	0.0552 (11)	0.0441 (10)	0.0137 (10)	0.0221 (9)	−0.0006 (9)
C13	0.0623 (12)	0.0805 (14)	0.0601 (13)	0.0194 (11)	0.0267 (10)	0.0038 (10)
C14	0.0560 (12)	0.0791 (14)	0.0490 (11)	0.0208 (10)	0.0130 (9)	0.0070 (10)
C15	0.0470 (10)	0.0664 (12)	0.0498 (11)	−0.0113 (9)	0.0157 (8)	−0.0135 (9)
C16	0.0535 (11)	0.0732 (13)	0.0557 (12)	−0.0040 (10)	0.0141 (9)	−0.0105 (10)
C17	0.0603 (12)	0.0911 (16)	0.0513 (11)	−0.0140 (11)	0.0161 (9)	−0.0092 (11)
C18	0.0684 (14)	0.0973 (18)	0.0617 (14)	−0.0247 (13)	0.0310 (11)	−0.0278 (13)
C19	0.0646 (13)	0.0787 (15)	0.0832 (17)	−0.0096 (11)	0.0370 (12)	−0.0239 (13)
C20	0.0565 (12)	0.0698 (13)	0.0672 (13)	−0.0063 (10)	0.0216 (10)	−0.0086 (11)
N1	0.0641 (10)	0.0628 (10)	0.0464 (9)	−0.0078 (8)	0.0193 (7)	−0.0086 (8)
N2	0.0627 (10)	0.0615 (10)	0.0540 (10)	−0.0048 (8)	0.0171 (8)	−0.0040 (8)
N3	0.0638 (10)	0.0638 (11)	0.0449 (9)	−0.0083 (8)	0.0151 (7)	−0.0043 (8)
N4	0.206 (3)	0.0672 (14)	0.0819 (15)	0.0173 (16)	0.0322 (16)	−0.0113 (13)
N5	0.1225 (16)	0.0619 (11)	0.0510 (10)	−0.0128 (10)	0.0238 (10)	−0.0003 (8)
N6	0.1076 (16)	0.0713 (12)	0.0516 (10)	0.0168 (11)	0.0311 (11)	0.0007 (9)
O1	0.1160 (14)	0.1135 (14)	0.0769 (11)	0.0135 (11)	0.0567 (11)	0.0013 (10)
O2	0.1504 (18)	0.1383 (16)	0.0511 (9)	0.0640 (14)	0.0312 (10)	0.0233 (10)

### Geometric parameters (Å, °)

**Table:**

C1—N2	1.318 (3)	C12—C13	1.371 (3)
C1—C2	1.431 (3)	C12—N6	1.471 (3)
C1—C7	1.489 (3)	C13—C14	1.375 (3)
C2—C3	1.399 (3)	C13—H13	0.9300
C2—C6	1.408 (3)	C14—H14	0.9300
C3—C4	1.386 (3)	C15—C16	1.381 (3)
C3—C9	1.488 (3)	C15—C20	1.384 (3)
C4—C8	1.428 (3)	C15—N1	1.425 (2)
C4—C5	1.441 (3)	C16—C17	1.387 (3)
C5—N3	1.333 (2)	C16—H16	0.9300
C5—N5	1.348 (3)	C17—C18	1.376 (3)
C6—N3	1.336 (2)	C17—H17	0.9300
C6—N1	1.365 (2)	C18—C19	1.372 (3)
C7—H7A	0.9600	C18—H18	0.9300
C7—H7B	0.9600	C19—C20	1.384 (3)
C7—H7C	0.9600	C19—H19	0.9300
C8—N4	1.142 (3)	C20—H20	0.9300
C9—C10	1.378 (3)	N1—N2	1.397 (2)
C9—C14	1.380 (3)	N5—H5A	0.8600
C10—C11	1.376 (3)	N5—H5B	0.8600
C10—H10	0.9300	N6—O2	1.216 (2)
C11—C12	1.362 (3)	N6—O1	1.222 (2)
C11—H11	0.9300		
			
N2—C1—C2	110.21 (17)	C13—C12—N6	118.86 (19)
N2—C1—C7	120.72 (18)	C12—C13—C14	118.58 (18)
C2—C1—C7	129.00 (18)	C12—C13—H13	120.7
C3—C2—C6	117.42 (18)	C14—C13—H13	120.7
C3—C2—C1	136.70 (18)	C13—C14—C9	120.68 (17)
C6—C2—C1	105.87 (16)	C13—C14—H14	119.7
C4—C3—C2	116.66 (17)	C9—C14—H14	119.7
C4—C3—C9	121.18 (17)	C16—C15—C20	120.50 (19)
C2—C3—C9	122.16 (17)	C16—C15—N1	120.44 (18)
C3—C4—C8	119.51 (18)	C20—C15—N1	119.04 (18)
C3—C4—C5	120.85 (17)	C15—C16—C17	119.3 (2)
C8—C4—C5	119.60 (19)	C15—C16—H16	120.4
N3—C5—N5	117.52 (18)	C17—C16—H16	120.4
N3—C5—C4	122.75 (19)	C18—C17—C16	120.4 (2)
N5—C5—C4	119.73 (18)	C18—C17—H17	119.8
N3—C6—N1	126.23 (17)	C16—C17—H17	119.8
N3—C6—C2	127.62 (17)	C19—C18—C17	119.9 (2)
N1—C6—C2	106.15 (17)	C19—C18—H18	120.0
C1—C7—H7A	109.5	C17—C18—H18	120.0
C1—C7—H7B	109.5	C18—C19—C20	120.5 (2)
H7A—C7—H7B	109.5	C18—C19—H19	119.7
C1—C7—H7C	109.5	C20—C19—H19	119.7
H7A—C7—H7C	109.5	C19—C20—C15	119.4 (2)
H7B—C7—H7C	109.5	C19—C20—H20	120.3
N4—C8—C4	178.2 (3)	C15—C20—H20	120.3
C10—C9—C14	119.01 (18)	C6—N1—N2	110.91 (15)
C10—C9—C3	120.08 (17)	C6—N1—C15	130.13 (16)
C14—C9—C3	120.91 (16)	N2—N1—C15	118.94 (16)
C11—C10—C9	120.98 (19)	C1—N2—N1	106.83 (16)
C11—C10—H10	119.5	C5—N3—C6	114.61 (17)
C9—C10—H10	119.5	C5—N5—H5A	120.0
C12—C11—C10	118.46 (18)	C5—N5—H5B	120.0
C12—C11—H11	120.8	H5A—N5—H5B	120.0
C10—C11—H11	120.8	O2—N6—O1	123.6 (2)
C11—C12—C13	122.28 (18)	O2—N6—C12	117.6 (2)
C11—C12—N6	118.84 (18)	O1—N6—C12	118.81 (19)
			
N2—C1—C2—C3	176.8 (2)	N6—C12—C13—C14	177.45 (19)
C7—C1—C2—C3	−6.2 (4)	C12—C13—C14—C9	0.1 (3)
N2—C1—C2—C6	−1.8 (2)	C10—C9—C14—C13	0.7 (3)
C7—C1—C2—C6	175.12 (18)	C3—C9—C14—C13	−178.96 (19)
C6—C2—C3—C4	−2.4 (2)	C20—C15—C16—C17	0.6 (3)
C1—C2—C3—C4	179.1 (2)	N1—C15—C16—C17	178.90 (17)
C6—C2—C3—C9	176.39 (16)	C15—C16—C17—C18	−1.2 (3)
C1—C2—C3—C9	−2.1 (3)	C16—C17—C18—C19	0.9 (3)
C2—C3—C4—C8	177.67 (19)	C17—C18—C19—C20	0.0 (3)
C9—C3—C4—C8	−1.1 (3)	C18—C19—C20—C15	−0.5 (3)
C2—C3—C4—C5	0.0 (3)	C16—C15—C20—C19	0.2 (3)
C9—C3—C4—C5	−178.80 (17)	N1—C15—C20—C19	−178.06 (17)
C3—C4—C5—N3	2.7 (3)	N3—C6—N1—N2	178.85 (17)
C8—C4—C5—N3	−174.99 (19)	C2—C6—N1—N2	−1.3 (2)
C3—C4—C5—N5	−176.71 (18)	N3—C6—N1—C15	0.7 (3)
C8—C4—C5—N5	5.6 (3)	C2—C6—N1—C15	−179.46 (17)
C3—C2—C6—N3	2.7 (3)	C16—C15—N1—C6	40.1 (3)
C1—C2—C6—N3	−178.33 (18)	C20—C15—N1—C6	−141.6 (2)
C3—C2—C6—N1	−177.13 (15)	C16—C15—N1—N2	−137.89 (18)
C1—C2—C6—N1	1.82 (19)	C20—C15—N1—N2	40.4 (2)
C3—C4—C8—N4	−45 (11)	C2—C1—N2—N1	1.0 (2)
C5—C4—C8—N4	132 (10)	C7—C1—N2—N1	−176.20 (16)
C4—C3—C9—C10	102.6 (2)	C6—N1—N2—C1	0.2 (2)
C2—C3—C9—C10	−76.2 (3)	C15—N1—N2—C1	178.58 (15)
C4—C3—C9—C14	−77.8 (3)	N5—C5—N3—C6	176.90 (17)
C2—C3—C9—C14	103.5 (2)	C4—C5—N3—C6	−2.5 (3)
C14—C9—C10—C11	−0.9 (3)	N1—C6—N3—C5	179.64 (18)
C3—C9—C10—C11	178.8 (2)	C2—C6—N3—C5	−0.2 (3)
C9—C10—C11—C12	0.2 (4)	C11—C12—N6—O2	4.2 (3)
C10—C11—C12—C13	0.7 (4)	C13—C12—N6—O2	−174.2 (2)
C10—C11—C12—N6	−177.6 (2)	C11—C12—N6—O1	−176.6 (2)
C11—C12—C13—C14	−0.8 (3)	C13—C12—N6—O1	5.1 (3)

### Hydrogen-bond geometry (Å, °)

**Table:**

*D*—H···*A*	*D*—H	H···*A*	*D*···*A*	*D*—H···*A*
N5—H5A···O2^i^	0.86	2.13	2.981 (3)	168
C14—H14···N2^ii^	0.93	2.61	3.529 (3)	168

Symmetry codes: (i) *x*, −*y*, *z*+1/2; (ii) −*x*+1/2, *y*−1/2, −*z*+1/2.

## References

[bb1] Bruker (2007). *SMART *and *SAINT* Bruker AXS Inc., Madison, Wisconsin, USA.

[bb2] Kamal, A. M., Atalla, A. A., Mohamed, T. A. A. & Geies, A. (1991). *Z. Naturforsch. Teil B*, **46**, 541–544.

[bb3] Quiroga, J., Alvarado, M., Insuasty, B., Moreno, R., Ravina, E., Estevez, I. & de Almedia, R. H. (1999). *J. Heterocycl. Chem.* **36**, 1311–1316.

[bb4] Sekikawa, I., Nishie, J., Tono-oka, S., Tanaka, Y. & Kakimoto, S. (1973). *J. Heterocycl. Chem.* **10**, 931–932.

[bb5] Sheldrick, G. M. (1996). *SADABS* University of Göttingen, Germany.

[bb6] Sheldrick, G. M. (2008). *Acta Cryst.* A**64**, 112–122. 10.1107/S0108767307043930 18156677

[bb7] Straub, A., Stasch, J.-P., Alonso-Alija, C., Benet-Buchholz, J., Ducke, B., Feurer, A. & Furstner, C. (2001). *Bioorg. Med. Chem. Lett.* **11**, 781–784. 10.1016/s0960-894x(01)00073-7 11277519

[bb8] Zhu, S.-L., Tu, S.-J., Li, T.-J., Zhang, X.-J., Ji, S.-J. & Zhang, Y. (2005). *Chin. J. Org. Chem* **25**, 987–990.

